# Mental States: A Key Point in Scam Compliance and Warning Compliance in Real Life

**DOI:** 10.3390/ijerph19148294

**Published:** 2022-07-07

**Authors:** Xin Wen, Liang Xu, Jie Wang, Yuan Gao, Jiaming Shi, Ke Zhao, Fuyang Tao, Xiuying Qian

**Affiliations:** 1 Department of Psychology and Behavioral Sciences, Zhejiang University, Hangzhou 310027, China; wenxin0301@zju.edu.cn (X.W.); xuliang_psy@zju.edu.cn (L.X.); wj_psy@zju.edu.cn (J.W.); ygao34@zju.edu.cn (Y.G.); shijiaming@zju.edu.cn (J.S.); 2 Ant Group, Shanghai 200120, China; zhaoke.zk@antgroup.com (K.Z.); fuyang.tfy@antgroup.com (F.T.)

**Keywords:** online fraud, scam compliance, warning design, mental states

## Abstract

The internet’s convenience and anonymity have facilitated different types of covert fraud, resulting in economic, mental, and social harm to victims. Understanding why people are deceived and implementing appropriate interventions is critical for fraud reduction. Based on the Bayesian brain theory, individuals’ mental states may be a key point in scam compliance and warning compliance. Fraud victims with different mental states may construct various hypotheses and explanations about the fraud they are exposed to, causing different cognition and behavior patterns. Therefore, we first conducted a semi-structured in-depth interview with online fraud victims to investigate the individual and social factors that affect victims’ mental states. Grounded theory analysis showed five core factors influencing scam compliance: psychological traits, empirical factors, motivation, cognitive biases, and emotional imbalance. Based on our findings of psychological processes and deception’s influential factors, we then designed warnings to inform victims of fraud, particularly for those involving novel types of scams. Tested on a real-life setting, our designed warnings effectively enhanced warning compliance, allowing more fraud victims to avoid financial losses.

## 1. Introduction

The growth and broad usage of the internet not only provides convenience to users but also raises the risk of fraud [1]. According to the FBI Internet Crime Complaint Center, the direct economic loss caused by online fraud in 2018 was as high as USD 2.7 billion [2]. This online fraud has a severe influence on individuals’ financial and overall well-being [3,4], adversely affecting the perception of interpersonal trust in the internet environment as well [5,6,7,8,9].

Currently, the types of scams have changed significantly and are markedly different from the phishing scams that have been primarily investigated so far. A slew of new deception schemes targeting mobile internet payment platforms has emerged with distinct features [10]. Primarily, real-time telephone or text interactions take place between the scammers and victims, causing the scammers to execute different fraud strategies based on the victim’s real-time response, resulting in more accurate and efficient fraud. Second, these scams have presented a wealth of diverse new ways to reach victims in unexpected ways. As a result, victims are often unaware of such scams.

These new features make it more challenging to detect scams and synchronously intervene. Since the interactions and transactions between scammers and victims usually done on separate platforms (chat platforms and payment platforms), there is no sufficient information and evidence to detect fraud on a single platform. To reduce the incidence of fraud, payment platform researchers generally deploy decision-aid tools, typically pop-up warnings for potentially risky transactions. However, barriers hamper the effectiveness of the warnings. During the transaction, the victim established a deep interpersonal relationship with the fraudster. In addition, because fraud is a low-probability event, victims tend to underestimate the risk of fraud [11,12]. Therefore, third-party pop-up warnings are not powerful enough to be alert when compared to “new friends” who offer “benefits”.

Moreover, as the difficult detection issues mentioned earlier, trading platform risk detection is not precise enough, resulting in high false positive rates. This creates a trade-off between effectiveness and disturbance, further increasing the difficulty of warning design. 

These resistances call for an investigation of why people fall prey to scams in these new types of scams, and warning intervention to help people avoid financial losses. To the best of our knowledge, no such combined studies are available.

A number of studies have been conducted to address why people fall victim to scams and lose money in the other type of scams, mainly focused on phishing. Research on fraud has focused on both the victim characteristics and mechanisms of fraud compliance. 

For the last thirty years, the characteristics of individuals sensitive to fraud have been a vital subject in several fields with shared concerns and discussion, such as psychology, criminology, and behavioral economics. Given the difficulty of directly contacting fraudsters, the majority of known information about scammers comes from descriptions of victims or rumors, making rigorous research and analysis of scammers restricted [13]. As a result, fraud research has concentrated on fraud vulnerability and compliance. Several systematic reviews have explored the impact of individual characteristics on internet fraud victimization based on consumer behavior, persuasion, decision making, and other fields related to deception [14,15,16,17,18,19,20,21,22,23,24]. The characteristics included age, nationality, culture, experience, psychological orientation, irrationality, and cognitive bias. These characteristics were considered primary, individual-level explanations for vulnerability to fraud [25,26,27]. Additionally, contextual factors such as time pressure were considered [17].

In addition, there are also concerns for studying the psychological mechanisms of fraud compliance [24,28,29,30,31,32,33,34]. Most of the mechanisms were based on the elaboration likelihood model (ELM; [35]), heuristic-systematic model (HSM; [36]), or deception detection theory. According to the staged model of trust, when evaluating trustworthiness, individuals were affected by a sample heuristic process unless the motivation and resources for systematic processing were sufficient [37]. The model shared similar conceptual cores with the elaboration likelihood and heuristic-systematic models. In ELM, individuals process information mainly in two ways: centrally or peripherally, which is analogous to systematic and intuitive processing in HSM. The central path refers to the careful assessment of information, i.e., refined processing, whereas the peripheral path evaluates using clues, i.e., the source of information, other than information. If the information recipient has the motivation and ability to perform refined processing, the central path will be used to process the information. Otherwise, the peripheral path will be taken [35]. Per the fraud detection model, individuals need to go through four processes to discover deception in information, including activation, which compares information clues in the environment with expectations about the clues. If there is a significant disparity between the clues and the expectations, the “abnormality” of the clue is activated. The next step is hypothesis generation, producing deceptive explanations for the aberrant clue, followed by hypothesis evaluation, assessing the significance of the clue, and determining whether the deceptive hypothesis of the clue should be considered. The final step is overall evaluation, which thoroughly assesses the deceptiveness of the judged object and determines whether the object is credible [30]. These four steps all involve the in-depth processing of fraud information and belong to refined processing [24].

Other studies have sought to establish a more detailed model that considers the factors influencing scam compliance [38]. The vulnerability model, for example, summarized the effect of visceral effects on scam compliance [39]. The suspicion, cognition, and automaticity model (SCAM) states that cognitive, preconscious, and automatic processes potentially lead to phishing deception [40]. Greenspan (2009) developed a four-factor causation model for vulnerable behaviors, which contained situational (e.g., time pressure or social pressure), cognitive, emotional, and personality (e.g., agreeableness) components and stated that the interactions among components affected scam compliance [41]. Norris, G., & Brookes, A. (2021) combined the interaction of emotional states and ELM [16]. They focused on how the interaction between accidental emotion (that is, the mood of an individual when receiving a scam message that is not related to a decision, such as when choosing to respond to a phishing email while feeling afraid of speaking at work) and overall emotion (that is, emotional states directly triggered by complaints embedded in fraudulent information (such as fear complaints)) affects personal decision making and processing of information when subjected to deceptive influence and put forward the viewpoint of maintaining emotion/repairing emotion [42,43] as a solution.

In summary, the previous literature on scam compliance mainly focused on two topics: (a) how individual differences affect scam susceptibility and (b) the mechanism of information processing in the scam. However, since fraud is a complicated reality issue, these two aspects interact. Individuals’ traits and emotional states may influence their information process. Previous studies have shown similar views. Norris, G., & Brookes, A. (2021) focus on the interaction effect of emotional states and ELM, providing further strategies to diminish scam compliance in light of environmental context factors [16]. Modic (2012) investigated the influence of state self-control and trait self-control on being deceived [19]. Research has found that individuals showed different mental states when involved in a scam [43], such as moods of expecting high rewards, sunk-cost concerns, or just in a state of great agitation. These mental states were situational factors that influenced the victims’ perception and cognition. According to the “Bayesian brain” theory, the brain makes probabilistic inferences about the world based on the input information and models and implements inferences for the targeted situation. Rather than passively following information from the outside world, the brain actively generates hypotheses about the world and uses hypotheses to explain the environment. In the course of the scam, the victims were actively constructing hypotheses and explanations about the scam scene, forming their specific mental state, which may have affected the participant’s perception of the entire event. As a result, the perception of the scam and warnings in the “controlled fantasy” scam scene were greatly different from those in the pure lab assessment. Thus, to better understand why victims are frauds and how victims perceive the pop-up warnings, it is necessary to have an overall insight into the psychological process of being deceived.

Therefore, the current study has two focuses. The first objective was to lay the groundwork for a better understanding of why people fall victim to scams and lose money in interactive mobile frauds using grounded theory methods. This qualitative method is useful to efficiently mine extensive and detailed information from the experiences of real participants, which is valuable in understanding the process of being deceived and the structure of influencing factors.

The second objective was to design effective warnings based on the analysis of psychological processes and influential aspects of fraud and to provide some insights into the warning design of current new deception schemes. Studies focusing on pop-up warnings in interactive scams have rarely been reported. Previous studies usually extend traditional warning design guidelines in human–computer interaction (HCI) to scam scenarios, focusing on phishing [44,45]. However, as overwhelming research points out that humans are the most important part of the HCI loop, in an intervention for deceived individuals, the detailed feedback and analysis of the perception of pop-up warnings from victims in real scenarios merit further investigation. Individual experiences and feedback can provide insight into warning design. Interviewing victims enables us to obtain participants’ real experiences and perceptions of the current warning and victims’ specific states when involved in scams. Furthermore, we conducted online testing in real life to assess the efficacy.

## 2. Study 1

### 2.1. Study Design

In Study 1, we conducted semi-structured, in-depth interviews with online fraud victims to understand why people fall prey to scams and lose money in interactive mobile scams, and we used grounded theory approaches to develop a structured model of scam compliance from the interview texts. Ethical approval was obtained prior to recruitment.

### 2.2. Participants

We conducted semi-structured interviews with 17 interviewees (2 males and 15 females) who had experienced being deceived at least once. Given the density and intensity of the information, we only included those who had a financial loss of more than CNY 100 in our study to ensure ample information was provided. The sample’s age range was 18–25 years, which is the typical internet user age [46]. Their educational background ranged from undergraduate to doctoral degrees. The types of deception included monetary deception through billing help, which is a kind of part-time job deception by asking victims to buy something online for the fraudster but not giving back their money or items purchased afterward, identity deception by impersonating friends and relatives, and investment scams, among others, and the amount of loss per scam varied from CNY 68 to 22,000 (more detailed demographic information is provided in Appendix A—Table A1). Participants received a reward of CNY 50 each. Informed consent was obtained from all participants.

### 2.3. Data Collection

Semi-structured, in-depth interviews were conducted with online fraud victims to better understand their psychological responses and cognitive perceptions during the fraud process, particularly how they react to pop-up warnings. An outline with open-ended questions guided the interview. Following the interview, the outline was modified based on an analysis of the interviewees’ corpus, and we present the final version of the outline here.

1. Please describe your overall experience of being deceived.
2. (Follow-up question) How did you determine that the current situation was not deceptive?
3. (Follow-up question) How did you determine the person whom you communicated with was believable?
4. (Follow-up question) In your view, which step played the biggest role in your victimization?
5. What did you think during the whole process of fraud?
6. (Follow-up question) What happened to your psychological state during the fraud?
7. Did the payment platform pop up with a warning when you paid? (If yes: how did you view the warnings at the time?)
8. Do you think you are a person who can be easily deceived? How do you come to this conclusion?

The interview lasted between 40 and 90 min, and the workplace was a quiet office.

### 2.4. Data Code and Analysis

We used Nvivo12 software (Melbourne, Australia) to analyze and code the data according to constructivist approach of Charmaz (2011) [47]. We first constructed the open coding (initial coding), axial coding, and selective coding (focused coding). The theory was then improved by comparison with pertinent ideas in the theoretical coding process. Three psychology graduate students coded the data constituted triangulation via multiple researchers [48,49]. The coders closed read, sentence-by-sentence analyzed, compared, and discussed areas of contradictions and disagreement before reaching a conclusion. For the saturation test, no additional main categories or core categories were developed during the coding of the 16th and 17th interview texts, indicating that the coding was saturated [47]. Specifically, no new nodes were added to the quotations in the 7th, 8th, 9th, 12th, 14th, 17th, and 18th interview texts.

#### 2.4.1. Open Coding

Open coding was conducted first. A total of 12,857 words were identified as relevant to the research topic in the open coding process. We conceived and categorized the original meaningful phrases without using any theoretical framework to get basic notions and categories. The interview texts of 17 respondents were coded sentence by sentence, and keywords with original expressions were maintained to the greatest extent feasible, resulting in 38 nodes, such as risk taking, underestimating risk, impulsiveness, and others (Table 1).

#### 2.4.2. Axial Coding

Then, in the axial coding process, we classified and compared different categories, extracting the main categories. We revealed 15 major categories, including risk-taking preference, risk perception, and trust, among others (Table 1).

#### 2.4.3. Selective Coding

The final procedure is selective coding, which involves analyzing the link between different main categories and establishing the “core category” that could regulate the category via repeated comparison. The experience element, for example, was divided into three major categories: safety knowledge, social experience, and operating experience. Selective coding yielded five core categories: psychological features, experience factors, motivation, cognitive biases, and emotional imbalance (Table 1).

#### 2.4.4. Theoretical Coding

**Figure 1:**
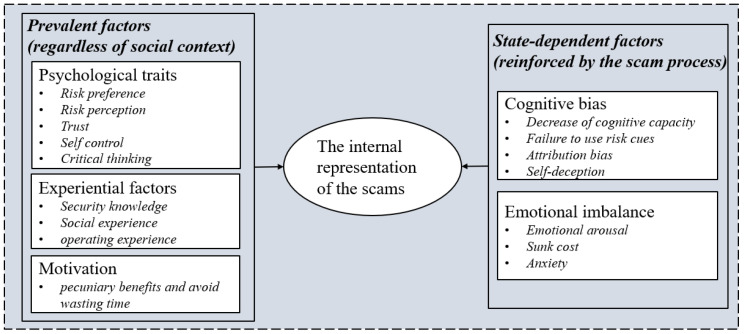
The influence factors of scam compliance.

Theoretical coding aims to find the relationship between the categories generated in the process of substantive coding by constantly comparing the established theories and relevant concepts. Compared with previous related theories, we found a relatively apparent structured relationship between these influential factors. All factors were separated into two categories: the prevalent factors that existed regardless of social context and the state-dependent factors reinforced by the scam process. The prevent factors contained psychological traits, which are the dispositional factors referred to in the previous research [17,41]; experiential factors, which are the equivalent categories of experiential factors in the SCAM [40]; and motivation, which is mentioned as visceral effects in the vulnerability model of scam compliance [39]. The state-dependent factors included cognitive bias and emotional imbalance. Cognitive bias is a similar concept that refers to victims making decisions based upon a heuristic analysis in line with a previous study [17,40,50]. Emotional imbalance refers to victims being in an abnormal emotional state when involved in the scam, which has some overlap with the emotional states in the four-factor causation model for vulnerable behaviors [41]. Together, these two components altered the victims’ psychological image of the current scam environment (Figure 1). 

### 2.5. Results and Discussion

#### 2.5.1. The Influence Factors of Scam Compliance

By integrating 18 interview texts, the core categories that emerged from the analysis were psychological traits, empirical factors, motivation, cognitive biases, and emotional imbalance. These five core categories together constituted the structure of factors influencing scam compliance. Among them, psychological traits, experience factors, and motivation were the individual intrinsic factor of the victims, whereas cognitive bias and emotional disorders were the state factors that were reinforced by the fraudsters in the fraud process.

Motivation, psychological traits, and experience factors are the factors related to the personal intrinsic dimensions of the victim. Personality traits are stable cognitive and behavioral patterns consistent in various contexts, including everyday life and fraud contexts. They have already become the individuals’ characteristics before individuals encounter the fraudster. Experience factors are victims’ social experience, knowledge about fraud, and experience that made them vulnerable to fraud. These experiences were also acquired in daily life. Motivation mainly includes two subcategories: pecuniary benefits and avoidance of wasting time. As victims expressed in interviews “Anyway, I prefer making a little money over doing nothing at all in my spare time”. Financial incentives and the motive to have something to do in their free time drove victims’ attention to fake “part-time job” messages and made them contact the scammers. The victims also showed attentional bias to visceral clues, which may be affected by the recent work environment in which individuals are engaged (i.e., their specific needs, such as lack of money) or more stable differences in primary motivations, such as the need for achievement, affiliation or influence needs [51].

The psychological traits related to susceptibility to scam compliance included five subcategories: risk preference, risk perception ability, trust, self-control, and critical thinking. Risk preference mainly included two subcategories: risk-taking and a high degree of openness to experience. Victims often have a high degree of risk acceptance or acceptance of new things, which conforms to the definition of risk preference [52]. The results indicated that victims tended to underestimate risks, which is consistent with the previous study indicating that people also tend to underestimate their vulnerability to phishing attacks [11]. The impulse and lack of patience expressed by the victim in the interview were related to the self-control category.

Similarly, participation in risky behavior has been seen as a vulnerability factor associated with online susceptibility to fraud in previous studies [53], and personality traits such as low self-control, sensation-seeking, and impulsiveness have all been linked to risky behavior across multiple domains [54]. Trust mainly included the victim’s trust in human nature and interpersonal trust. Some victims said they believed that there would not be so many swindlers to deceive others. They believed in people’s kindness and expressed their trust in human nature. Some victims also said they would easily believe in what others say, even a joke, showing a high level of trust in interpersonal communication. Trust in human nature refers to victims’ appraisal and perception of the broader population’s honesty, whereas interpersonal trust refers to the behavioral pattern of believing in the statements of others. Our results supported the correlation between trust and vulnerability to fraud [34,55,56,57], but there were some opposite findings in previous studies [58]. One possible explanation could be that trust reduces perceived risk [59,60]. In addition, the victims sometimes said “I had to believe in him though I truly sometimes suspected he was a liar” after they were cheated out of a large amount of money. They also tended to search for information to confirm their thoughts and stick to their choices when others told them they were being cheated. These results reflected confirmation bias and a lack of truth-seeking and critical thinking processes and were hence summarized into the category of insufficient critical thinking.

Experience factors included three subcategories: safety knowledge, social experience, and operating experience. Victims usually had certain misconceptions about online security or a lack of anti-fraud knowledge, resulting in poor online security knowledge and causing unsafe online behaviors, such as leaking private information. Victims also stated that they lacked social experience or that their experience with online shopping or transferring was limited, making them duped. Previous research has also found that online experience can reduce the likelihood of responding to phishing emails [33,34,61]. According to channel expansion theory [62], experience and knowledge improve perceiving and digesting of subtle information in a conversation. Research in the field of phishing also found that experience with email or instant messaging can not only help with detecting subtle clues to deception [63], but it can also make one more confident in discovering deceptive information [64]. 

After being involved in a scam, victims frequently find themselves in cognitive bias and emotional imbalance, either spontaneously or induced by scammers. Some victims claimed to have experienced a feeling of sleepiness and distraction, as well as cognitive deficits and difficulty with systematic processing while being deceived. Furthermore, exerting time pressure was also commonly used by scammers, constantly urging victims to follow the steps they provided to complete the payment quickly. This kind of distraction, drowsiness, and time pressure collectively caused the victim to fail to be alert.

As a consequence of failure in vigilance, victims made decisions based upon a heuristic analysis in line with a previous study [17,40,50], specifically referring to a variety of cognitive biases, such as habituation and ignoring risk cues in our analysis. Much research evidence points to individuals preferring economical over effortful information evaluation [65,66]. In addition, victims showed a sense of attribution bias. Due to this bias, some victims were accustomed to internal attribution or spontaneously reasoned uncertainties. On the other hand, scammers used tricks similar to “that’s all your fault” words to induce victims to misattribute. In addition, a proclivity for self-deception was noted. This process was meaningful when people with risk aversion to take risks because self-deception and information rejection can provide self-protection or self-enhancement [1,67].

Emotional imbalance refers to victims being in an abnormal emotional state when involved in the scam. For example, for the multi-round profitable scam, the scammer gave a bait task with high-profit margins in the first round. The victim received a high bonus and entered a positive emotional state of high arousal. Furthermore, their cost–benefit consideration drives them to picture a pleasant scenario of getting a large amount of wealth and leads to visceral or peripheral information processing. However, in the other types of scams, the victim’s emotional state might be the opposite. Victims who have lost part of their principal in the scam and have sunk-cost considerations are often in a negative emotional state of worry, anxiety, or even panic. People who are depressed or have the insufficient mental strength to overcome impulses are more likely to fail in self-control [68], leading to escapism and nonrational behaviors.

Finally, in the context of being deeply involved in the scam, victims engaged in a “distorted” mental state that was expressed as “senseless” by the victims. This “distorted” mental state further caused the rash action to become an obviously unreasonable decision, and victims then used self-deception to reduce their uncertainty about the decision. As a result, they fell into an interlocking trap, act, defend, and act again (similar to cognitive dissonance), which strengthened the intensity and commitment of their actions and ultimately led to a deeper trap.

#### 2.5.2. Insights on the Design of Anti-Fraud Warnings

The interview results relevant to the warning proception show the following intriguing results: 52.94% of the victims encountered warnings provided by the transaction platform during the payment process, 35.29% of the victims skipped the warnings altogether, 29.41% of the victims raised doubt about why there were warnings, and among them, 11.76% asked the scammer and 23.53% self-rationalized the warning. This finding indicated that although some of the victims saw the warnings and even had adequately understood them, at the moment of payment, the victims had no intention of carefully thinking about the risk of payment mentioned in the warning and wholly ignored the warning. When asked victims why they did not carefully think about the risk of payment mentioned in the warning, they said they just thought it was a general warning not targeted to them or thought it was a bug in the platform. In summary, they did not realize they were in a scam, and thus, they were in a mental state that did not acknowledge the concept of scams. This cognition affected their goal-oriented attention and made them create an incorrect explanation for the clues. Thus, unlike other studies, we argued that the application of warning design in the scam context could not be separated from the understanding of how individuals are defrauded because they share the same implicit internal representation system at the moment. This representation system influences individuals’ perception of the pop-up warning. Only if we understand the mental states of the victims in real-life can a detailed and reasonable warning be formulated.

These findings further provided insight into how people react to pop-up warnings in scam scenes. In the interview, we found that the victim was already in a completely different mental state when facing warnings compared with an ordinary context, in line with previous studies [69]. This mental state would profoundly affect the victim’s top-down attention and affect the victim’s awareness of the pertinence of the warning, and largely determine the effectiveness of the warnings. Previous frameworks on warning design, such as the communication-human information processing (C-HIP) model [70] and the human-in-the-loop security framework [71], were typically based on the communication-processing model, which describes how a message from a source is sent to a receiver and then triggers some behavior. Researchers have explored various meaningful and influential dimensions, such as personal variables, intentions, capabilities, communication delivery, communication processing, and others [71]. However, the receivers’ mental states at the moment the warning signals pop up merit further study.

**Figure 2:**
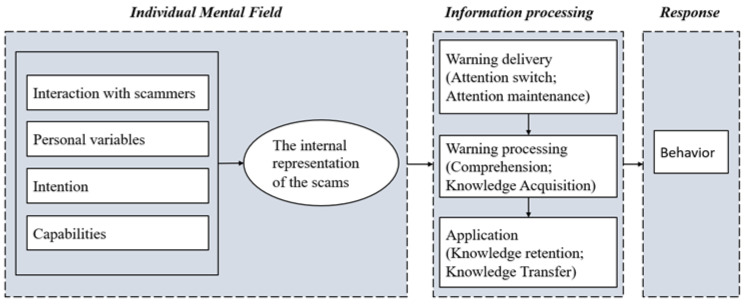
A scaffold for the process of the warning.

To clarify, we proposed a warning processing schematic diagram in the scam context (Figure 2). The diagram contains three domains: individual mental state, information processing, and behavior. The individual mental state has four major components corresponding to the factors that influence scam compliance, founded in study1: personal variables, intentions, capabilities, and interactions with scammers. These four components collectively affected the warning receivers’ current internal representation of the scams and formed the mental state. This mental state is the outcome of individuals’ active cognitive construction of the context, and it affects the receiver’s goal-oriented attention to stimuli. Then, there is a filtering process. If the stimulus is consistent with the individuals’ expectations, it goes to the information processing step. Otherwise, the stimulus is ignored because the individual considers the information to be targeted elsewhere. In detail, the victims felt that they were not in the scam in the current scam scenario. This awareness made them think the current warning had nothing to do with them. It was just a general warning, and they skipped the warning directly.

To cope with these resistances, we presented a guideline for conducting effective warnings based on understanding the victims’ mental states in the scam context. The findings implicate that the first and the most crucial point is to capture user attention and raise awareness that the warning pop out is related to their current trade. Because victims self-reported cognitive bias and were self-convinced that the warning was regularly presented and irrelevant to currency trading. Next, since victims are often in an emotional state or devoted to communicating with scammers, the warning should be in heuristic strategies. The anchor strategies, clear behavioral guidance, and the negative loss-based description of consequences may be effective. In addition, a description of the fraudulent strategies is required, as victims frequently lack detailed knowledge of the scams. We further designed warnings based on our findings and conducted an online experiment to test the warnings to implement into real-life practice.

## 3. Study 2

In this section, we tested the effectiveness of two warnings designed based on our findings in a real online transaction environment. We assumed that the warning designed according to the victims’ mental-state-oriented findings could achieve better results than the current warning used in real life.

### 3.1. Methods

#### 3.1.1. Platform and Participants

The online experiment was conducted on the Alipay platform. Alipay is the largest third-party online payment platform in China, with more than 100 million transactions per day. The Intelligent Risk Control Engine (IRCE) is a decision-aid tool co-created with Alipay and China’s Ministry of Public Security to prevent online fraud [72]. In pursuit of a better anti-fraud effect, IRCE assists ongoing efforts to implement risk control in fraud detection and pop-up warnings, which obtain users’ informed consent. We partnered with the Alipay platform to conduct anti-fraud research. The participants were anonymous users identified as being involved in risky transactions. The platform would conventionally pop up the warnings about unsafety transactions to guard their property. In the current experiment, more than 4 million users were presented with the baseline warning and 43,015 users were presented with warning 1, and 153,470 users were presented with warning 2. Considering the nature of the field experiment, we could not provide additional informed consent to participants. As users are in real risky transaction scenarios, additional informed consent may lead to users’ misjudgment of the current riskiness and affect their trust in the warning, which affects the security of transactions subsequently and the reproducibility of the experiment. The experiment was not expected to pose any additional risks to participants. Instead, it may have the defensive benefits of shielding participants from the actual risk of fraud. The experiment was ethically approved by the ethics committee (PS-2019058) and passed the audit by the legal department of Alipay. 

#### 3.1.2. Materials

It should be noted that the IRCE system also faces difficulties in fraud detection, as mentioned in the introduction, which results in a high rate of false alarms. To avoid an uncomfortable payment experience for users who are not at risk and comply with legal requirements, the effectiveness/non-disturbance trade-off must be considered, which means there are some restrictions. For instance, if it is not accurate enough, the warning cannot use words such as “The counterparty account is abnormal” or “Your trading partner is a new account” because of user privacy considerations.

To cope with these restrictions, we used nudges to design detailed warning terms combined with the guidelines of our warning processing model. The most important part of the warning is the attention acquisition part, which should occupy the user’s primary focus and make the users feel that the warning is valuable. Therefore, warning 1 and warning 2 were designed using the same guidelines, which enhance attention first, remind them of deception tricks specifically, and then state the consequences.

**Figure 3:**
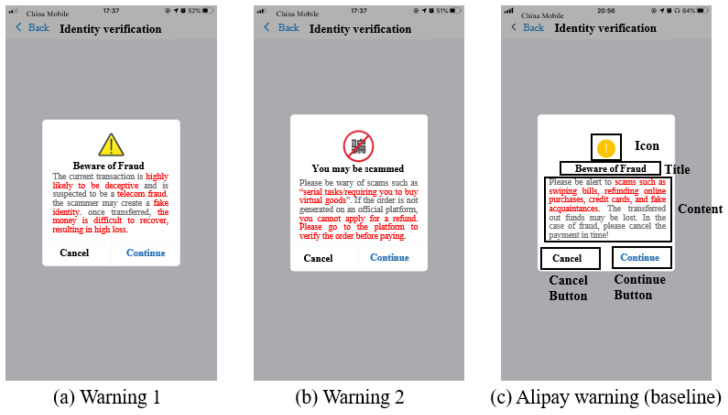
Materials of Warnings.

In warning 1, we used the sentence “The current transaction is highly likely to be deceptive and is suspected to be a telecom fraud” in the first line to increase user attention by informing the user directly that he is at risk and arouse awareness of the scam through anchoring. Then, we used the sentence “the scammer may create a fake identity,” corresponding to our finding that victims usually lacked essential safety knowledge. Usually, they were not aware of such scams unless they received a clear reminder. We expected to create friction [73] to confront the scam. Third, we used the sentence “once transferred, the money is difficult to recover, resulting in high loss” at the end of the warning, which aimed to remind the victim of the possible negative consequences and arouse the victim’s negative perceptions. For the icon, we used a combination of yellow triangles and exclamation points to enhance attention and arouse hazard perceptions by using traffic warning metaphors (Figure 3a).

In warning 2, we used the sentence “You may be scammed” as the title to directly tell users that he/she may be involved in a scam and create an alert. Second, we used the sentence “Please be wary of scams such as “serial tasks/requiring you to buy virtual goods” (because virtual goods cannot be returned while most of the users may not have known this regulation) to explain the possible tricks to the users. Third, we used the sentence “If the order is not generated on an official platform, you cannot apply for a refund” to expound on the consequences. Then, we offered alternatives to users to give their behavior direction with the sentence “Please go to the platform to verify the order before paying”. In this way, we hope to alert confused users by providing specific descriptions about what they might encounter and safe behavior guidelines to confront scammers. As for the icon, we used a banned metaphor, which is commonly used in traffic warnings, along with the word “fraud” to convey the concept “No fraud” (Figure 3b).

In the baseline warning, which is the current Alipay warning, the title is “Beware of Fraud”, and the content used the sentence “Please be alert to scams such as swiping bills, refunding online purchases, credit cards, and posing as an acquaintance. The money could be lost. In the case of fraud, please cancel the payment in time!”. The icon is a commonly used icon for reminding people (Figure 3c).

#### 3.1.3. Procedure

An online A/B test was conducted to test effectiveness. If a transaction was detected as potential online fraud by the IRCE system, there would pop out a warning. We tested warning 1, warning 2, and the baseline warning for two months. Each warning was tested in a random group. Warning 1 was tested in small random numbers of users (N = 43,015 Alipay users), and warning 2 was tested in a random wider group (N = 153,470 Alipay users). The baseline was tested in the remaining users (approximately 4 million Alipay users).

In addition, effectiveness and non-disturbance were evaluated in terms of the case rate and the pass rate. The case rate represents the ratio of the fraud case, and a lower-case rate represents a better warning effect. The pass rate represents the likelihood of the user choosing “continue to pay” when the current transaction is not risky, measuring the degree to which the warning interferes with the user’s payment process. A higher pass rate represents a lower disturbance, especially false alarm conditions. The calculation formulae are rendered thus:

*Case Rate = The number of online fraud cases after warning controls/The number of warning controls* (1)

*Pass Rate = Correct rejection rate = The number of correct rejections/The number of warning controls where the * *number of correct rejections represents the safe transaction, and the users ignored the warning.* (2)

### 3.2. Results

**Figure 4:**
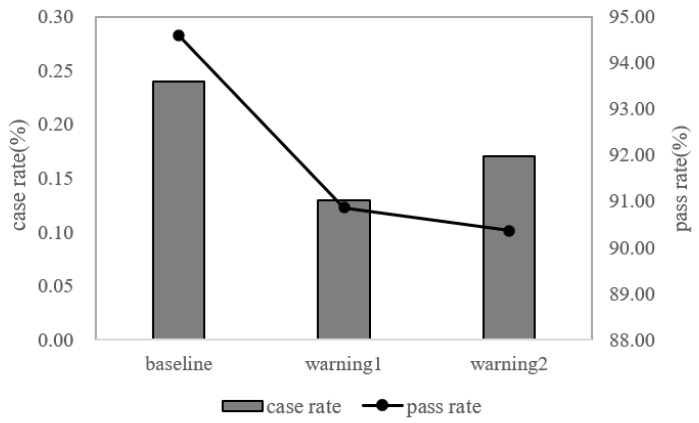
The results of the online A/B test. The bar graph represents case rate and the line graph represents pass rate.

The results show that the improved warnings performed better than the baseline warnings on case rates. For warning 1, the case rate reduced from 0.24 (baseline) to 0.13. The improvement was 62.3%, and for warning 2, the case rate reduced from 0.24 (baseline) to 0.17, an improvement of 45.8% (Figure 4). The chi-square test showed that the case rates of warning 1 and warning 2 were significantly lower than the baseline warning (χ^2^ = 20.92, *p* < 0.01; χ^2^ =27.08, *p* < 0.01). 

For the pass rate, though we detected a significant difference between warning 1 and the baseline group (χ^2^ = 2418.42, *p* < 0.01), as well as warning 2 and the baseline group (χ^2^ = 9917.68, *p* < 0.01), all warnings had a pass rate of over 90%, which is considered satisfactory and acceptable commercially (90.36% vs. 94.58%; 90.86% vs. 94.58%, the latter is the baseline warning. Thus, these results supported that the warning designed based on the current optimized model can reduce scam compliance to some extent. Given the large victims group size, a small improvement in case rate can bring considerable financial benefits for the users.

## 4. General Discussion

This study started with the basic question of why people fall victim to scams and lose money in interactive mobile scams. Based on an analysis of interview texts, the substantive and theoretical coding led to five distinct core categories predominantly involved in scam compliance that constituted the structure of scam compliance. The structure combined two components: (a) the prevalent factors existing regardless of the social context and (b) the state-dependent factors reinforced by the scam process. These two components, to some extent, corresponded to the findings of how personal factors affect scam susceptibility [1,4,16,17,19,20,21,22,23,24,25,26,27] and information processing in scams [24,28,29,30,31,32,74,75]. In our study, personal factors corresponded to psychological traits, empirical factors, and motivation, and the information processing about scams corresponded to cognitive biases and emotional states.

Qualitative analysis is widely used in research on scam compliance [50] and benefits user experience research [76]. The grounded theory relies on a standardized process to derive concepts and models or theories from the original materials without a priori assumptions. In our study, the model provided a good foundation for constructing the scam compliance structure. Indeed, it should be noted that grounded theory relies on the researchers’ insights and deep understanding of the relevant concepts to some extent, whereas strict coding procedures can help to compensate for concerns about subjectivity. In addition, it was recommended to find similarities and differences compared with extant grounded theory. In summary, suitable use of grounded theory methods might shed light on the holistic understanding of scam compliance.

Furthermore, the present study designs effective warnings based on the analysis of psychological processes and influential aspects of fraud, as well as provides some insights into the warning design of current new types of deception schemes. Our research indicated that in the warning design process, not only the various elements mentioned in HCI but also the internal mental states of warning recipients should be considered. This finding is not only in line with Bayesian brain theory but can also be found based on social cognitive psychology. The traditional S (stimulus)-O-R (response) in the cognitive approach insists on participants behaving as an active organism (O) that intervenes between the stimulus and the response. Furthermore, the assumption of O evolved to a wider scope from the early view, extending from perception, selective attention, memory, and context effects to schemas, scripts, and other complex structures.

In summary, it was generically considered a euphemism for the mind or alternative as a focus on internal representational states, as the concept of mental state in this study. Internal states not only mediate the relationship between the stimulus and its behavioral consequences but also attenuate the perception of the previous stimulus to control which stimulus to target and which to ignore. Thus, the new point of view was represented as O-S-O-R, and individuals’ active cognitive construction of the environment subsequently became an essential part of the behavior process [77]. 

Therefore, we recommend that in the warning design, it is better to focus on the relationship between elements and victims’ internal mental representations to the greatest extent, increasing users’ attention. Only when the user considers the current pop-up warnings correlated with themselves and deserve attention may the warning be effective. In addition, we used field research to test our warnings in a real scam compliance situation with a huge group of participants with a high degree of external validity.

There remain some limitations in this study. First, in study 1, there were much more women than men (88% women) in our sample. Although, according to Telecom Network Fraud Governance Report [10], the scam type relevant to the current study harms women more frequently than men, with a 7:3 female preponderance, there is a limitation to generalizing our results to men. Second, to achieve higher ecological validity, we conducted an online A/B test to examine our model, but it was difficult to strictly control variables in the field research and gain insight into the relationship between mental states and variables. Confounding variables, such as the length of the warnings or the description of the severity of the consequences, may further affect the effectiveness of the warnings. Thus, a more delicate laboratory experiment that could evoke similar mental states in a real context could be conducted to investigate the interaction of warning elements and the user’s mental state. Third, study 2 only focused on a few categories of the structure. A more detailed analysis of more factors may provide additional targeted information for intervening scams, such as the warning targeted to the remission of emotional disorders or personalized warning based on individuals’ dispositional traits. We are unsure whether such warnings are still effective in other domains, such as the use of hazardous chemicals, where better warning strategies may exist. Furthermore, future studies may delve into better strategies. Moreover, a combination of the design model with new forms of warnings, such as eye-catching pictures, can be considered in future work.

## 5. Conclusions

The current study conducted a semi-structured in-depth interview with online fraud victims to investigate the individual and social factors that affect victims’ mental states in a real scam context. The results showed five core factors influencing scam compliance: psychological traits, empirical factors, motivation, cognitive biases, and emotional imbalance, according to qualitative analysis. Based on the findings of psychological processes and deception’s influential factors, we then designed warnings to inform victims of fraud, especially those engaging in novel sorts of scams. When tested in a real-world scenario, the warnings designed based on the findings show significantly higher warning compliance, with a 54.05% improvement, assisting more fraud victims in avoiding financial losses. 

## Figures and Tables

**Table 1:** Coding results of the fraud influencing factors.

Selective Coding	Axial Coding	Open Coding	Frequencies for Axial Codes
Psychological traits	Risk preference	High openness to experience, High risk-seeking (High risk-taking)	4
	Risk perception	Risk underestimation	10
	Trust	High interpersonal trust, High faith in human nature	13
	Self-control	High impulsiveness, Lack of patience	8
	Critical thinking	No questioning when having doubts, Rash acceptance of fraudsters’ explanation	11
Experiential factors	Security knowledge	Problematic security knowledge, Lack of security knowledge, Lack of information protection awareness	15
	Social experience	Young age, Being naive, Lack of contact with strangers, Lack of social experience	12
	Operating experience	Little experience with the exact kind of business (e.g., refund in Amazon), Little experience with online shopping, Little experience with transfers	13
Motivation	Avoid wasting time	Having spare time	8
	Pecuniary benefits	Being induced by money as bait	10
Cognitive bias	Decrease in cognitive capacity	Being distracted, Fatigue, Being under time pressure	7
	Failure to use risk cues	Failure to notice risk cues, Misidentify a warning as a less risky one	16
	Attribution bias	Incorrect attribution, Internal attribution	4
	Self-deception	Self-convinced	3
Emotional imbalance	Emotional arousal	Excitement	3
	Sunk cost	Being eager to make up sunk cost	7
	Anxiety	Worry, Anxiety (Nervousness), Panic	10

## Appendix A

**Table A1:** Sociodemographic information of each participant.

Index	Gender	Age	Age When Scammed	Financial Loss (CNY)
1	Male	26	24	598
2	Female	22	22	8000
3	Female	24	20	1850
4	Female	20	19	1800
5	Female	23	20	900
6	Female	23	19	299
7	Female	23	22	600
8	Male	22	18	1000
9	Female	24	23	22,000
10	Female	21	18 & 20 (scammed twice)	4400 & 7400 & 13,000
11	Female	23	18 & 21 (scammed twice)	8000 & 3000
12	Female	21	21	1000
13	Female	22	22	13,000
14	Female	24	22	6000
15	Female	21	18	2400
16	Female	24	23	10,000
17	Female	21	20	2000
